# Synthesis of a Novel P/N-Triazine-Containing Ring Flame Retardant and Its Application in Epoxy Resin

**DOI:** 10.3390/polym16070871

**Published:** 2024-03-22

**Authors:** Yi Yu, Junlei Chen, Anxin Ding, Changzeng Wang, Yunfei Wang, Ling Yang

**Affiliations:** 1 School of Materials Science and Engineering, Wuhan University of Technology, Wuhan 430070, China; 1998.yy07@gmail.com (Y.Y.); czwang@whut.edu.cn (C.W.); tufuachuan1@163.com (Y.W.);; 2 Institute of Advanced Material Manufacturing Technology, Wuhan University of Technology, Wuhan 430070, China; axding@whut.edu.cn

**Keywords:** reactive flame retardant, bio-based flame, mechanical properties, retardant properties

## Abstract

To meet the environmental protection and flame retardancy requirements for epoxy resins (EPs) in certain fields, in this study, a novel triazine-ring-containing DOPO-derived compound (VDPD), derived from vanillin, 2,4-Diamino-6-phenyl-1,3,5-triazine, and 9,10-dihydro-9-oxa-10-phosphaphenanthrene-10-oxide (DOPO), was synthesized using a one-pot method. Flame-retardant epoxy resin (FREP) was prepared by adding various ratios of VDPD to EP and curing with 4,4-diaminodiphenylmethane (DDM). The curing behavior, thermal stability, mechanical properties, and flame-retardant properties of the FREP were examined in various tests. According to the results, when the amount of VDPD added to the EP increased, the glass transition temperature of the FREP decreased linearly, and the flame-retardant properties gradually improved. With a 0.4 wt.% P content, the vertical burning rating of EP/DDM/VDPD-0.4 (according to the theoretical content of VDPD) reached the V-0 level, and the LOI value reached 33.1%. In addition, the results of a CCT showed that the peak heat release rate (PHRR) of EP/DDM/VDPD-0.4 decreased by 32% in comparison with that of the EP. Furthermore, compared with those of the EP, the tensile strength of EP/DDM/VDPD-0.4 decreased from 80.2 MPa to 74.3 MPa, only decreasing by 6 MPa, and the tensile modulus increased. Overall, VDPD can maintain the mechanical properties of EP and effectively improve its flame-retardant properties.

## 1. Introduction

Epoxy resin (EP) is a well-known thermosetting polymer with excellent physical, chemical, and electrical insulation properties, thus having wide applications in various fields, such as the industrial, construction, electronic, automotive, and aerospace fields [1,2,3,4]. However, EP is highly flammable, easy to burn, and a fire hazard, severely limiting its application. Thus, improvement in the fire safety of EP is highly desired [5,6].

Initially, researchers developed halogen-containing flame retardants to improve the flame-retardant properties of EP. However, during combustion, halogen-containing flame retardants release toxic gases, which are harmful to the environment and human health. This prompted studies on halogen-free flame retardants. Among them, phosphorus-based flame retardants have attracted widespread attention because of their excellent flame-retardant properties and benign nature towards the human body. Among the various phosphorus-based flame retardants, 9,10-dihydro-9-oxa-10-phosphaphenanthrene-10-oxide (DOPO) [7] is one of the most important ones. Due to DOPO’s biphenyl structure and P-H bond, it possesses various advantages, such as an excellent thermal stability, excellent antioxidant behavior, highly flame-retardant efficiency, and low toxicity. In the condensed phase, DOPO can promote char formation, and, in the gas phase, it can release •PO as a free radical scavenger, thus providing highly flame-retardant properties to EP. Furthermore, DOPO is suitable for producing a wide variety of flame retardants due to its ability to react with a variety of active groups [8]. For example, XU et al. [9] synthesized a phosphorus-containing flame retardant (CTP-DOPO) using, 4-aminophenol, DOPO, hexachlorocyclotriphosphazene, and p-hydroxybenzaldehyde as raw materials. The EP system doped with 10.6 wt.% CTP-DOPO showed high flame retardancy in a vertical combustion (UL-94) test, being rated the V-0 level in UL-94, and it had a limiting oxygen index (LOI) of 36.6%. Yang et al. [10] combined DOPO, N-(4-hydroxyphenyl) maleimide (HPM), and cyanuric chloride as raw materials to synthesize a flame retardant (DOPD-TMT). With the addition of 14 wt.% DOPO-TMT, the EP/DOPO-TMT-0.1 sample achieved the V-0 level in the UL-94 test, and its LOI increased to 36.2%. It is obvious that the addition of flame retardants consisting of DOPO derivatives to EP is a reasonable method for improving the flame-retardant properties of EP. However, EP requires a large amount of DOPO derivative flame retardants to obtain excellent flame retardancy, but that would damage the overall performance of EP. Therefore, it is necessary to enhance the flame-retardant efficiency of DOPO derivatives to reduce the negative impact of the addition amount.

In order to further enhance the flame-retardant efficiency of flame retardants with DOPO derivatives, the N element has been introduced to improve flame-retardant efficiency through cooperation with the P element. It is well known that nitrogen-containing compounds decompose into nitrogen-containing small molecules and that the released gas dilutes the concentration of combustible gas. The released gas can also expand the char layer and reduce the transfer rate of heat and substances. Additionally, phosphorus-containing compounds quench free radicals and promote the formation of a phosphorus-containing char layer, thereby improving the flame-retardant efficiency [11]. Therefore, according to the above flame-retardant mechanism, an effective method is to introduce compounds with strong stability and high nitrogen content to enhance the flame-retardant efficiency of DOPO-derivative flame retardants. Among the compounds with high nitrogen content are benzoguanidine, melamine, and ammonium polyphosphate (APP). However, melamine and APP have poor compatibility with EP, whereas benzoguanidine has good compatibility with EP, high thermal stability, and high nitrogen content. Liu et al. [12] synthesized heterocyclic compounds (BDP) with active functional groups by using 2,4-Diamino-6-phenyl-1,3,5-triazine (DPT) and DOPO as raw materials. When adding 5 wt.% BDP, the LOI of vertical combustion was 35.2%, thus achieving the V-0 level.

The global shortage of petroleum resources and the call for green policies have attracted many researchers to use renewable materials to prepare flame retardants. Therefore, the discovery of natural materials that can be utilized to synthesize effective flame retardants is critical in order to protect the environment and ensure sustainability [13,14,15,16,17,18,19,20]. Recently, bio-based materials including vanillin [21], eugenol [22], lignin [23], resveratrol [24], and cinnamaldehyde [25] have become increasingly popular and are being commonly used as raw materials for flame retardants. Among them, vanillin (VN) is a suitable choice. Vanilla extract originates from lignin, and it is most commonly used in the food industry as a disinfectant, antibacterial, and palate-genic agent, as well as a curing agent [26]. For example, J. Cao et al. [27] employed a one-pot method using vanillin, guanine, and DOPO as raw materials to prepare a bio-based reactive flame retardant (VFD). EP/VFD-7.5, with 7.5 wt.% VFD, reached the -0 level in UL-94, with the LOI increasing to 34.5%. Sun et al. [28] prepared a flame retardant designated as TFD by employing tyrosine, furfuraldehyde, and DOPO. When the TFD dosage was 6 wt.%, the LOI value of EP/TFD-6 was 32.4 vol%, and it achieved the V-0 level in UL-94, thus demonstrating high flame retardancy. To develop flame-retardant agents, the use of bio-based materials and DOPO is critical.

As part of this study, flame-retardant epoxy resin was prepared by using an innovative novel triazine-ring-containing DOPO-derived compound, synthesized from DOPO, VN, and DPT as reactive flame retardants, and DDM as a curing agent. In addition, the chemical structure of VDPD and the thermal stability, mechanical properties, and flame-retardant properties of the epoxy resin with VDPD were analyzed using various devices, and the flame-retardant mechanism is discussed.

## 2. Materials and Methods

### 2.1. Materials

Diglycidyl ether of bisphenol A (DGEBA, E-51: epoxide value 0.51 mol/100 g) was acquired from Nantong Xingchen Synthetic Material Co., Ltd. (Nantong, China). Anhydrous ethanol and toluene were obtained from Chengdu Kelong Chemical Co., Ltd. (Chengdu, China). Vanillin (VN) was purchased from Shanghai Yuan Ye Biotechnology Co., Ltd. (Shanghai, China). 9,10-dihydro-9-oxa-10-phosphaphenanthrene-10-oxide (DOPO) and 4,4-diaminodiphenylmethane (DDM) were purchased from Shanghai Macklin Biochemical Co., Ltd. (Shanghai, China). 2,4-Diamino-6-phenyl-1,3,5-triazine (DPT) was purchased from Wuhan Lanbai Pharmaceutical Chemical Co., Ltd. (Wuhan, China).

### 2.2. Synthesis of VDPD

**Figure 1:**
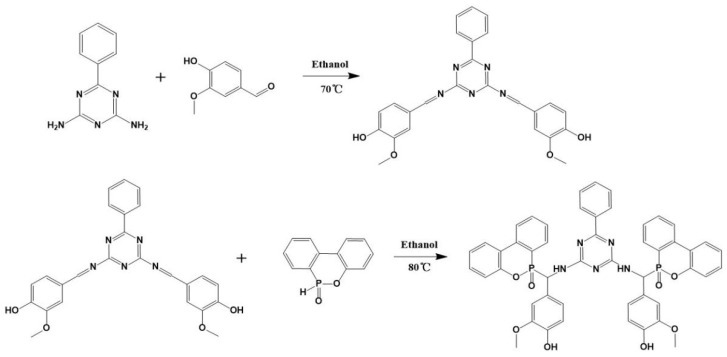
Synthesis of VDPD.

**Figure 2:**
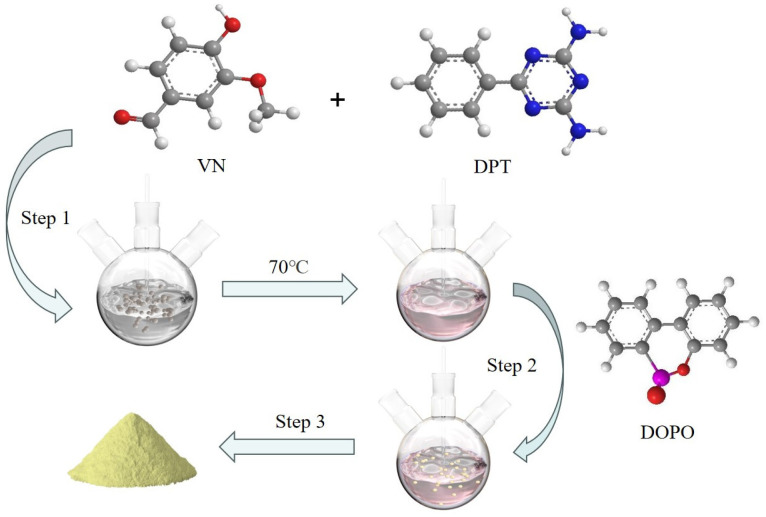
Operation steps.

Figure 1 depicts the synthesis method of VDPD, which was carried out by using a one-pot method. DPT (7.5 g, 0.04 mol), vanillin (12.16 g, 0.08 mol), and anhydrous ethanol (200 mL) were placed in a 500 mL, three-neck, round-bottom flask fitted with a condenser, stirrer, and thermometer, and the reaction was carried out at 70 °C for 6 h. Then, DOPO (17.22 g, 0.08 mol) was added to the mixture and stirred, and the reaction was allowed to continue for 24 h at 80 °C. Afterwards, the reaction mixture was allowed to cool to room temperature. The obtained precipitate was filtered, followed by washing with hot water (80 °C) and toluene. Subsequently, the obtained product was dried in a vacuum oven at 80 °C for 24 h to remove the residual solvent, yielding a light yellow solid product (yield: 86%). The operation steps are shown in Figure 2.

### 2.3. Preparation of Flame-Retardant EP

Flame-retardant epoxy resin (FREP) was prepared via the thermal curing reaction between VDPD, DDM, and EP. The proportions of the different reagents are given in Table 1 (the phosphorus weight percentage was calculated based on the theoretical amount of VDPD). First, after adding VDPD to EP, the mixture was heated and stirred at 80 °C for 10 min until it became transparent. Next, DDM was added and stirred for 10 min, before degassing under vacuum to remove bubbles. Finally, the mixture was cured at 100 °C for 2 h after being poured into a mold, followed by further curing at 150 °C for 4 h, and then, it was slowly cooled to room temperature. VDPD flame-retardant epoxy resin samples were obtained, and they were named EP/DDM/VDPD-X (X is the mass fraction of the P content, calculated according to the theoretical dosage of VDPD). The preparation method of pure epoxy resin was the same as that of the VDPD flame-retardant epoxy resin, but VDPD was not added, and the obtained sample was named EP/DDM.

### 2.4. Characterization

#### 2.4.1. Structure Characterization of VDPD

An American Nicolet 6700 infrared spectrometer (Waltham, MA, USA) was used to measure the Fourier Infrared Spectroscopy (FTIR) spectra in the range of 400–4000 cm^−1^. The VDPD and char residue samples were detected after KBr palletization.

The ^1^H NMR and ^31^P NMR spectra were measured using a Bruker AV 400 NMR spectrometer (Bruker, Karlsruhe, Germany) with DMSO-d6 as the solvent for further confirmation of the VDPD structure.

C, H, and N elemental analyses(EA) of VDPD were performed using a Vario EL cube CHNSO elemental analyzer (Elemental, Frankfurt, Germany).

#### 2.4.2. Curing Behavior and Thermal Stability Analysis

A Perkin-Elmer DSC 4000 (PE, Waltham, MA, USA) differential scanning calorimeter was used to carry out DSC measurements. The test conditions during the measurement were as follows: a heating rate of 5, 10, 15, and 20 °C/min and a heating temperature range of 50–300 °C under a nitrogen atmosphere.

A thermogravimetric analysis (TGA) was performed using a thermogravimetric analyzer STA2500 (Netzsch, Selb, Germany). The samples were heated from 25 to 800 °C under N_2_ conditions at a heating rate of 10 °C/min.

#### 2.4.3. Mechanical Properties Analysis 

Tensile strengths were obtained using an Instron 5967 device (Instron, MA, USA). According to the GB/T 1040.2-2006 test standard, the test sample gauge length was 50 mm, and the tensile rate was 2 mm/min. Five specimens were tested for each sample, and the average values are listed [29].

Izod unnotched impact strengths were determined using an XJJD-50 device (Xinma, Chengde, China). According to the GB/T 1843-2008 test standard, the test spline size was 80 × 10 × 4 mm^3^. Five specimens were tested for each sample, and the average values are listed [30].

A dynamic mechanical analysis (DMA) was performed using a specimen with dimensions of 60 × 10 × 3 mm^3^ and a DMA Q800 (TA, New Castle, DE, USA) apparatus in the three-point bending mode at a heating rate of 5 °C/min from 30 to 250 °C with a constant frequency of 1 Hz. 

#### 2.4.4. Flame Retardance and Combustion Behavior Analysis

The material was tested for limiting oxygen index (LOI) values according to ASTM D2863 by using a JF-3 oxygen index meter (Jiangning, Nanjing, China). The test spline size was 100 × 6.5 × 3 mm^3^. For each sample, the measurement was carried out five times, and the average values are listed [31].

A vertical combustion (UL-94) test of the material was carried out according to ASTM D3801 by using an NK8017A instrument (Nklsky, Dongguan, China); the test spline size was 130 × 13 × 3 mm^3^, and each sample was measured five times [32].

The combustion behavior of the material was tested using an FTT cone calorimeter (FTT, East Grinstead, UK) according to ISO 5660-1, maintaining a test spline size of 100 × 100 × 3 mm^3^ and an external heat flux of 35 kW/m^2^. Each sample was measured three times, and then, the results were averaged [33].

#### 2.4.5. Morphology and Chemical Analysis of Char Residues

The morphology of the combustion char residues was analyzed via scanning electron microscopy (SEM) using a Zeiss Ultra Plus scanning electron microscope coupled (Zeiss, Oberkochen, Germany) and with an energy-dispersive X-ray (EDX) microanalysis system, which was employed for the elemental analyses of C, O, and P.

Laser Raman spectroscopic (LRS) measurements were performed using a LabRAM Odyssey (Horiba, Paris, France) laser Raman spectrometer in the range of 600–2000 cm^−1^ with an excitation wavelength of 633 nm.

#### 2.4.6. Analysis of Pyrolysis Behavior

An analysis of the pyrolysis products of VDPD was carried out by using pyrolysis gas chromatography/mass spectrometry (Py-GC/MS) employing an Agilent 19091S-433 (Agilent, CA, USA) GC-MS. The inlet temperature was 40 °C, helium was used as the carrier gas, and the sample was cleaved at 500 °C.

## 3. Results and Discussion

### 3.1. Structural Characterization of VDPD

**Figure 3:**
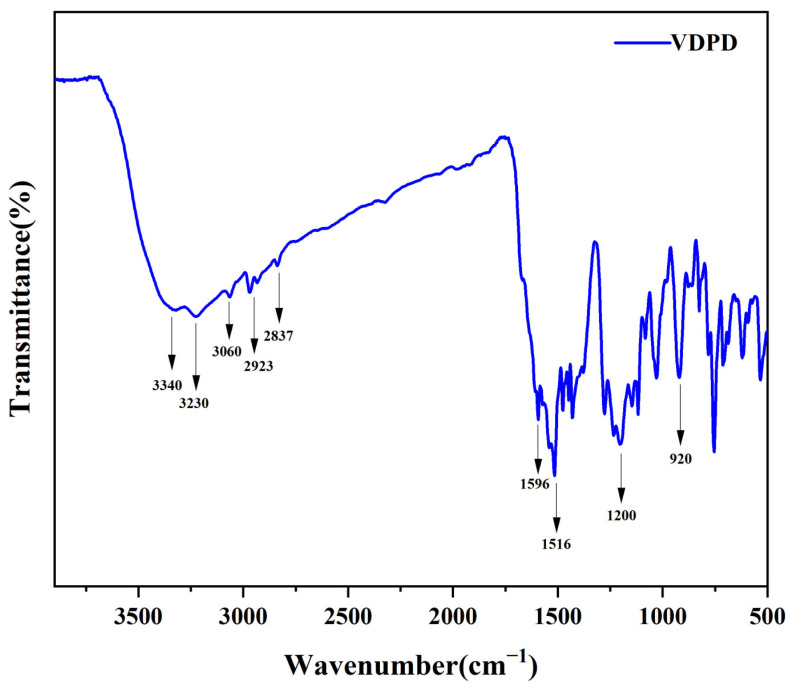
FTIR of VDPD.

**Figure 4:**
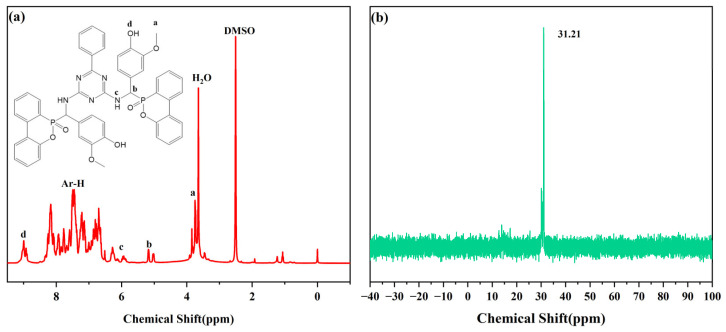
The ^1^H NMR spectra of VDPD (**a**) and ^31^P NMR spectra of VDPD (**b**).

FT-IR and NMR were used to characterize the structure of the flame-retardant VDPD. Figure 3 illustrates the infrared spectrum of VDPD. The spectrum displays absorption peaks at 3230, 2923, and 2830 cm^−1^, which correspond to the stretching vibrations of -OH, -CH3, and -CH2- bonds, respectively. The absorption peak at 3340 cm^−1^ can be attributed to the N-H group present in VDPD, and the presence of a benzene ring is indicated by the absorption peak at 3060 cm^−1^. The absorption peaks at 1596 cm^−1^ and 1516 cm^−1^ correspond to the presence of P-N bonds and the skeletal vibration of the benzene ring in VDPD, respectively, whereas the peaks at 1200 cm^−1^ and 920 cm^−1^ correspond to P=O and P-O-C bonds. The presence of absorption bands corresponding to P-N, P-O, and P-O-C proves the successful synthesis of VDPD. Figure 4 presents the ^1^H and ^31^P NMR spectra of the VDPD flame retardant. The peak at 8.98 ppm can be attributed to the chemical shift of active -OH, while 5.91–8.33 ppm corresponds to the chemical shift of aromatic hydrogen [28,34,35]. The chemical shift from 5.0 ppm to 5.24 ppm corresponds to C-H, while the shift of 3.73 ppm is attributed to -OCH3. The chemical shift of N-H is 5.76–6.04 ppm. Moreover, the presence of a signal at 31.3 ppm in the ^31^P NMR [36,37] indicates the presence of phosphorus-containing phenyl groups.

Furthermore, for further verification of the accuracy of the VDPD structure, an elemental analyzer was used for quantitative analyses of C, N, and H in VDPD, and the test results are summarized in Table 2. According to the theoretical chemical structure of VDPD, the highest percentage was found for element C, at 66.29%, followed by elements H and N, at 7.89% and 4.43%, respectively. The percentages of elements C, N, and H as per the CHN analysis were 66.26%, 7.74%, and 4.582%, respectively. Based on the above-described results, it could be concluded that the target product VDPD was synthesized as per the prediction.

### 3.2. Analysis of Curing Behavior

**Figure 5:**
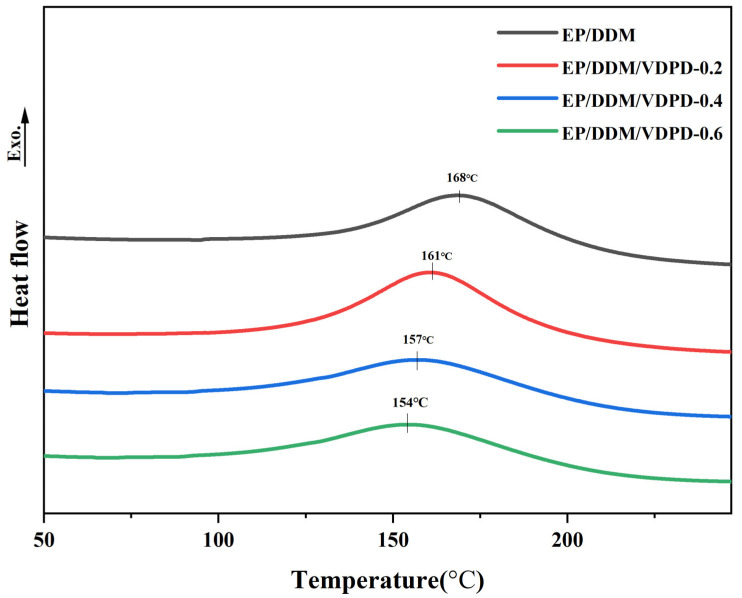
DSC curves of EP/DDM and EP/DDM/VDPD at a heating rate of 10 °C/min.

Differential scanning calorimetry (DSC) was used to investigate the curing behavior of the EP. As shown in Figure 5, each DSC curve presents only one exothermic peak. The exothermic peak of the EP was attributed to the addition reaction between the epoxy group and the amino group. The ring-opening reaction between the epoxy group, the amino group, and the hydroxyl group could be ascribed to the exothermic peak of the FREP. The peak temperature of EP/DDM was 168 °C, and, with the gradual addition of VDPD, the peak temperature of EP/DDM/VDPD-0.6 decreased to 154 °C, which indicates that VDPD can react with epoxy groups and improve the curing reaction activity of the curing system. For accuracy, the apparent activation energy was calculated using the more accurate Starink method [38,39]. The equation is as follows:

(1) $$\ln\left(\frac{\beta_{i}}{T_{\alpha ,i}^{1.92}}\right)=\;\mathrm{const}\;-1.0008\left(\frac{E_{\alpha }}{RT_{\alpha }}\right)$$

In the formula, *T_α,i_* is the equivalent conversion temperature at different heating rates, subscript *α* is the conversion (K), *β_i_* is the heating rate (°C/min), R is the gas constant, and *E_α_* is the apparent activation energy.

**Figure 6:**
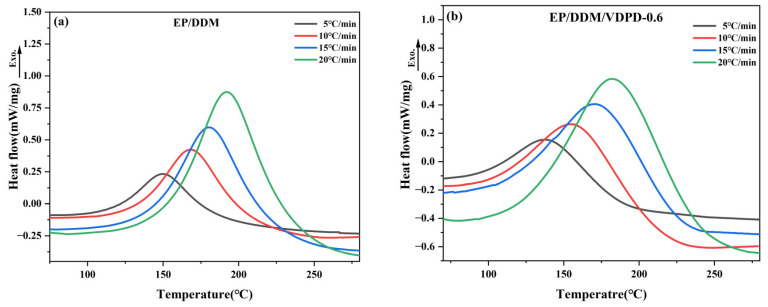
DSC curves of EP/DDM (**a**) and EP/DDM/VDPD-0.6 (**b**) at different heating rates.

**Figure 7:**
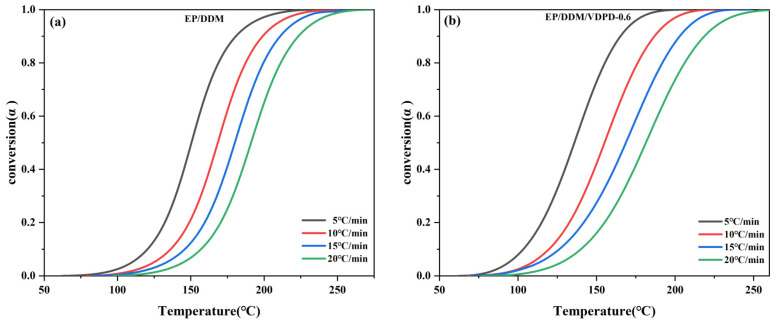
Plots of conversion α vs. temperature of EP/DDM (**a**) and EP/DDM/VDPD-0.6 (**b**) at different heating rates.

Based on the DSC curve at different heating rates shown in Figure 6, the relationship between *α* and *T* can be obtained by calculating Equation (2), as shown in Figure 7.

(2) $$\alpha =\frac{1}{\Delta H_{0}}\int_{0}^{t}(\frac{dH}{dt})dt$$

In the formula, Δ*H*_0_ is the total heat release (J/g); *t* is time (s); *α* is the conversion; *H* is the heat released at a certain time (J/g).

Different *α* values are selected from Figure 7 (*α* values are selected with a point interval of 0.10, *α* = 0.10, 0.20, 0.30……) (*T_α,i_*). According to Equation (1), *E_α_* is calculated according to the Starink equation, and *E_α_* is obtained by plotting the slope of the fitting line of ln (*β_i_*/*T_α,_*_i_^1.92^) and 1/*T_α_*.

**Figure 8:**
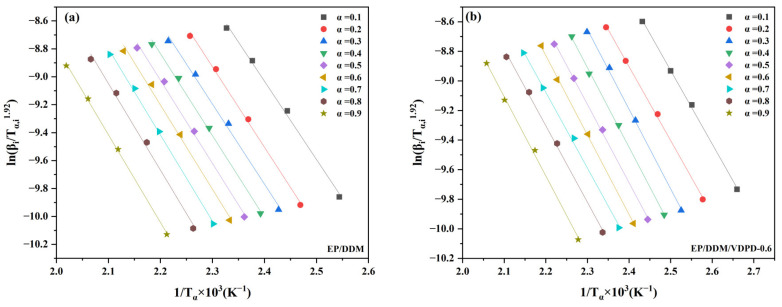
Starink plots of EP/DDM (**a**) and EP/DDM/VDPD-0.6 (**b**) at different conversion rates.

**Figure 9:**
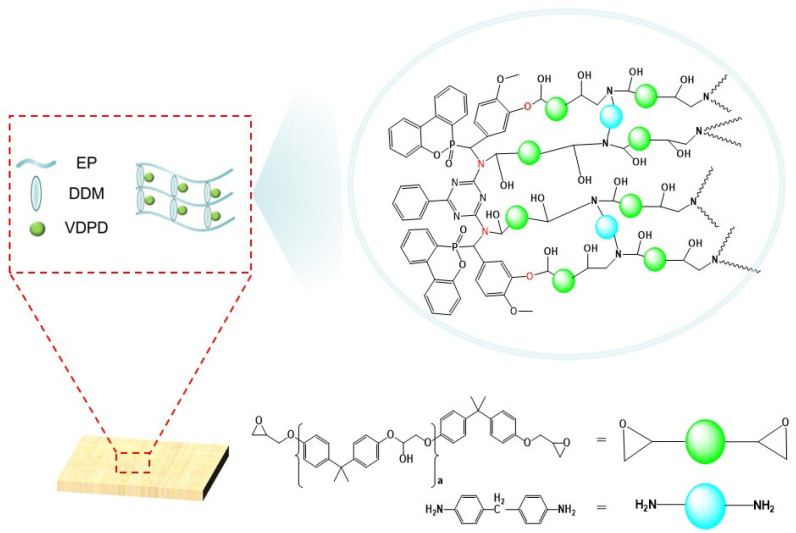
The possible crosslinking pattern of EP/DDM/VDPD.

Figure 8 shows a good linear fit. As can be seen in Table 3, the *E_α_* apparent activation energy range of EP/DDM is 46.58–52.25 kJ/mol, and the average *E_α_* is 49.51 kJ/mol; the *E_α_* range of EP/DDM/VDPD-0.6 is 41.37–44.56 kJ/mol, and the average *E_α_* is 43.10 kJ/mol. This suggests that the addition of VDPD resulted in a lower *E_α_* in the present curing reaction. Therefore, VDPD improves the curing activity of the curing system, which proves that the active hydrogen of VDPD reacts with the epoxy group. This possible cross-linking mode of EP/DDM/VDPD is indicated in Figure 9.

### 3.3. Dynamic Mechanical and Mechanical Properties

**Figure 10:**
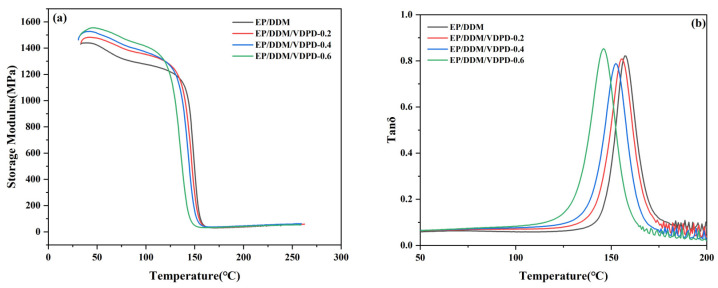
Storage modulus (**a**) and tan δ (**b**) curves of EP/DDM and EP/DDM/VDPD.

To illustrate the impact of VDPD on the EP thermodynamic properties, DMA was used to study EP/DDM and EP/DDM/VDPD. Figure 10 shows the curves of the storage modulus and the tan δ of the fully cured EP/DDM and EP/DDM/VDPD as a function of temperature. υe could be computed from the theory of rubber elasticity: υe = E’/3RT, where E’ represents the storage modulus at 40 °C above T_g_, R denotes the gas constant, and T stands for the thermodynamic temperature. The data are summarized in Table 4.

In Figure 10, it can be clearly seen that all the tan δ curves show a single peak, indicating high compatibility between VDPD and EP. The storage modulus of all EP/DDM/VDPD samples at 50 °C is higher than that of EP/DDM, indicating that VDPD can improve EP stiffness. The main reason for this is the introduction of an aromatic structure. However, with the increase in the VDPD content, the T_g_ value shows a decreasing trend, from 158 °C to 146 °C, suggesting that VDPD influences the EP T_g_. Both the stiffness and cross-linking density of the EP network affect the EP T_g_. On the one hand, the introduction of a large number of groups hinders the formation of a three-dimensional network structure, leading to a decrease in T_g_. The significant steric effect of the relatively large VDPD molecule may negatively affect the curing system’s cross-linking density. On the other hand, due to the low reactivity of some imino groups and hydroxyl groups with epoxy groups relative to DDM, some groups do not react with EP, resulting in a decrease in the cross-linking density. Due to VDPD’s steric hindrance and low reactivities, EP/DDM’s cross-linking density decreases from 3600 mol/m^3^ to 2996 mol/m^3^.

**Figure 11:**
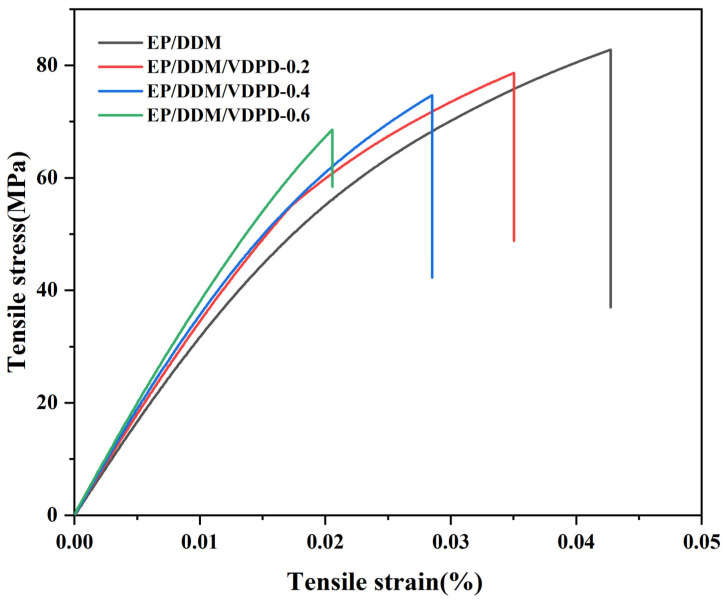
Stress–strain curves of EP/DDM and EP/DDM/VDPD.

The effect of VDPD addition on the mechanical properties of the EP was evaluated in a tensile test and an impact test. Figure 11 shows the stress-strain curves of EP/DDM and EP/DDM/VDPD. As per Table 5, the addition of VDPD had little effect on the mechanical properties. The tensile strength of the unmodified EP was 80.2 MPa, and, with a gradual increase in the VDPD content, the tensile strength of EP/DDM/VDPD-0.4 decreased to 74.3 MPa, reducing by 7%. On the one hand, the reason for this may be related to the triazine group and benzene ring, which inhibit the displacement between molecules; on the other hand, when VDPD participates in the curing reaction of EP, due to the low activity of some groups and the absence of solidification with the EP, the cross-linking density decreases, resulting in a reduction in tensile strength. However, the tensile modulus gradually increased with the addition of VDPD, from 3077 MPa to 3490 MPa, and the impact strength did not change much.

### 3.4. Thermal Stability

**Figure 12:**
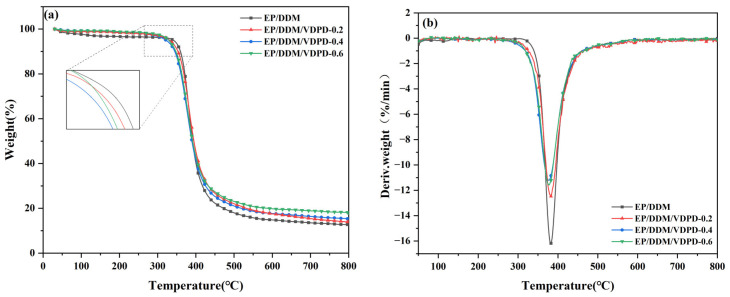
TG (**a**) and DTG (**b**) curves of EP/DDM and EP/DDM/VDPD.

The thermal stability of VDPD on the EP was evaluated by conducting a TGA in a nitrogen atmosphere, and the relevant results are summarized in Table 6. Thermogravimetric and differential thermogravimetric curves of the cured epoxy resin are depicted in Figure 12. As shown in Figure 12a, for EP/DDM, the highest 5% weight loss temperature (T_5%_) and the temperature at the maximum decomposition rate (T_max_) were 343 °C and 383 °C, respectively. With the addition of VDPD, there was a gradual increase in the P content, and the T_5%_ and T_max_ of EP/DDM/VDPD decreased gradually. The main reason for this phenomenon is that the thermal stability of the O-P=O bond in VDPD is lower than that of the C-C bond in cured epoxy resin [40]. The presence of only one DTG peak for all cured epoxy resins, as indicated in Figure 12b, suggests that there was only one thermal weight loss stage. Additionally, the maximum decomposition rate (R_max_) of the EP/DDM/VDPD curing agent decreased significantly in comparison to that of the EP/DDM curing agent; this indicates that VDPD addition improves the stability of EP at high temperatures. Moreover, the char residual content (800 °C) of EP/DDM/VDPD increased significantly from 12.22% to 21.0%. Thus, with the increase in the VDPD content, the residual char content increased significantly, which indicates that the addition of VDPD had a significant effect on the formation of char residues. In summary, although the introduction of VDPD accelerates the early decomposition of EP, it can improve the formation of char, increase the stability of EP at high temperatures, and inhibit combustion, thereby improving flame-retardant properties.

### 3.5. Flame Retardancy and Combustion Behavior

Basic tests for studying flame-retardant properties are the LOI and UL-94 tests, where the LOI represents the combustibility of the sample, and UL-94 indicates the extinguishing speed of the sample without interference. As per Table 7, the epoxy resin was flammable, having an LOI value of only 24.4% without a flame retardant. After the addition of VDPD, the LOI value of EP/DDM/VDPD-0.2 increased to 29.5%, while adding only 0.2 wt.% P content [41]. With the increasing addition of VDPD, the LOI increased from 29.5% to 34.5%. During the vertical combustion process, the EP had to be manually extinguished, as the flame spread to the fixture, though it did not produce any liquid droplets. The results from the vertical combustion test changed with the gradual addition of VDPD. The sample showed a distinct natural extinguishing phenomenon with the addition of only 0.2 wt.% phosphorus content, reaching a level of V1. When the phosphorus content was increased to 0.4 wt.%, the prepared sample easily reached the V-0 level. This demonstrates that, even at low phosphorus levels, VDPD could provide excellent flame-retardant properties to EP. The joint action of the triazine group and phosphaphenanthrene groups in VDPD is the primary reason behind this effect.

**Figure 13:**
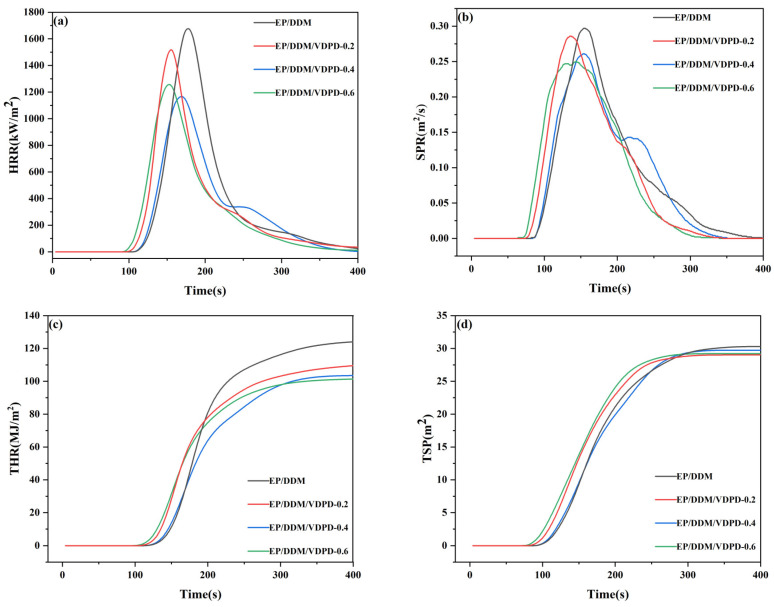
HRR (**a**), SPR (**b**), THR (**c**), and TSP (**d**) curves of EP/DDM and EP/DDM/VDPD.

Cone calorimetry was used to evaluate the combustion performance of EP/DDM and EP/DDM/VDPD [42]. Figure 13 presents variation curves of the heat release rate (HRR), total heat release (THR), smoke production rate (SPR), and total smoke production (TSP) with time. Other relevant data, such as the peak of the heat release rate (PHRR), average yield of CO (av-COY), average yield of CO_2_ (av-CO_2_), the average effective heat of combustion (av-EHC), and the char residue yield, are listed in Table 8.

The PHRR value of EP/DDM, as depicted in Table 8, was 1679 kW/m^2^, and the PHRR values decreased for all PREPs with the addition of VDPD. When the P content was raised to 0.4 wt.%, the PHRR of EP/DDM/VDPD-0.4 reduced by 32%, relative to that of EP/DDM, reaching a value of 1167 kW/m^2^. However, a further increase in the P content to 0.6 wt.% resulted in an increase in the PHRR. The introduction of a large number of functional groups that hinder the formation of a three-dimensional network structure is one of the reasons for the increase in the PHRR. Furthermore, as a result of their significant spatial effect, the cross-linking density of the curing system can be adversely affected by relatively large VDPD molecules. All PREPs had lower THR values than EP/DDM, which had a THR value of 124 MJ/m^2^. With an increasing VDPD content and a simultaneously increasing P content from 0.2 wt.% to 0.6 wt.%, the response value of the THR decreased from 111.4 MJ/m^2^ to 101.8 MJ/m^2^. Moreover, these results demonstrate a joint action of the phosphaphenanthrene groups and triazine groups, which reduced the burning degree of the FREP effectively.

The gas-phase combustion degree of combustible gases and the effective average combustion heat of flame retardants are characterized by av-EHC, which can be used to demonstrate the flame-retardant properties of VDPD. The av-EHC value of the EP was 39.70 MJ/kg, as can be seen in Table 8. With an increase in the P content to 0.4 wt.%, the av-EHC value of the FREP decreased from 39.70 MJ/kg to 33.2 MJ/kg, a decrease of 16%, thus demonstrating a gas-phase flame-retardant effect of VDPD. In addition, compared to the char residual content of the EP, a significant increase in that of EP/DDM/VDPD-0.6 was observed, from 10.3% to 18.3%, an increase of about 77%.

The SPR represents the amount of smoke produced during combustion. The peak of EP/DDM/VDPD-0.6 was at 0.249 m^2^/s, compared to 0.296 m^2^/s for the EP/DDM, a decrease of 16%. As shown in Figure 13d, with the increase in the VDPD content, the TSP decreased slightly, from 0.311 m^2^ to 0.29 m^2^, a decrease of about 6%. Increasing the VDPD content also resulted in an increase of approximately 60% for the av-COY of EP/DDM/VDPD-0.6 compared with that of the EP, increasing from 0.1356 kg/kg to 0.2164 kg/kg, though av-CO_2_ gradually decreased. As a result, when the VDPD content was increased, the incomplete combustion of EP/DDM/VDPD led to the thermal decomposition product of EP/DDM/VDPD, which inhibited the further oxidation of the combustible products of the epoxy resin to CO_2_, thereby resulting in a rapid increase in the COC peak. Based on the above results, VDPD can significantly increase the fire resistance and flame retardancy of EP.

These results suggest a gas-phase flame-retardant effect of VDPD, which can inhibit the combustion of flammable gases in the gas phase. The free radical quenching effect of phosphaphenanthrene groups and the nitrogen-containing flame-retardant gas dilution effect of triazine groups are the main reasons behind the gas-phase flame-retardant effect of VDPD. The use of VDPD can effectively promote the charring of EP and suppress the formation of combustible debris. During combustion in the condensed phase, a significant increase in residual char occurs due to the presence of the phosphaphenanthrene group in the VDPD structure, which can produce phosphates, polyphosphates, and other phosphates as a result. These substances and the residual char form a continuous protective layer, which acts as a cover and prevents EP combustion. Thus, the THR, PHRR, and av-EHC of the FREP were reduced due to the combined effects of the phosphaphenanthrene and triazine groups of VDPD.

As can be seen in the results of the LOI, UL-94, and cone calorimetry tests, the excellent flame-retardant properties of VDPD endowed excellent flame retardancy to the cured epoxy resins when the P content was low.

### 3.6. Morphology and Chemical Analysis of Char Residue

**Figure 14:**
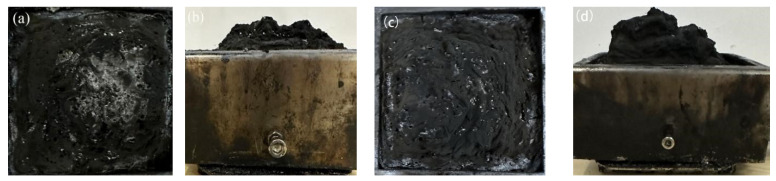
Digital images of the char residues from (**a**,**b**) EP/DDM and (**c**,**d**) EP/DDM/VDPD-0.6.

To determine the condensed-phase flame-retardant mechanism of VDPD, the morphology and chemical structure of the char residues after the CC test were investigated using SEM, EDX, FTIR, and Raman spectroscopy. Firstly, after the cone calorimeter test, as shown in Figure 14a,b, the char residue amount of EP/DDM was very small, and the structure was loose, indicating that the EP could not form a continuous and stable char layer during combustion. After the introduction of flame-retardant VDPD, as shown in Figure 14c,d, the char layer of EP/DDM/VDPD-0.6 expanded, the char residues increased significantly, and a continuous char layer formed.

**Figure 15:**
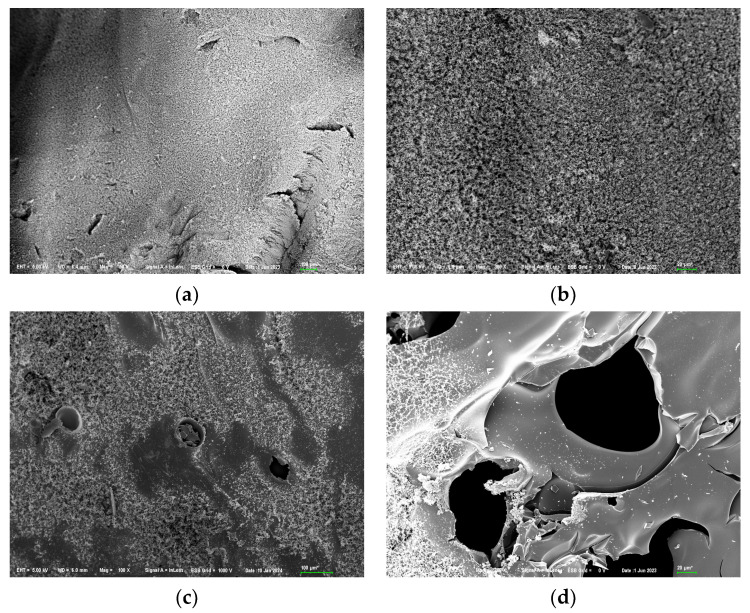
SEM images of the char residues from (**a**,**b**) EP/DDM and (**c**,**d**) EP/DDM/VDPD-0.6.

**Figure 16:**
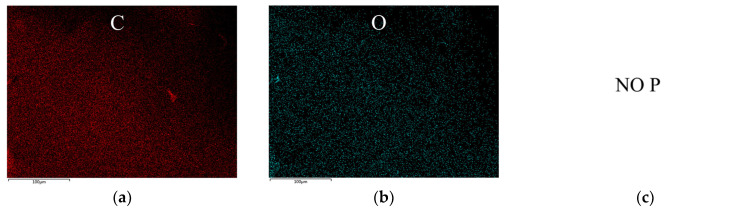

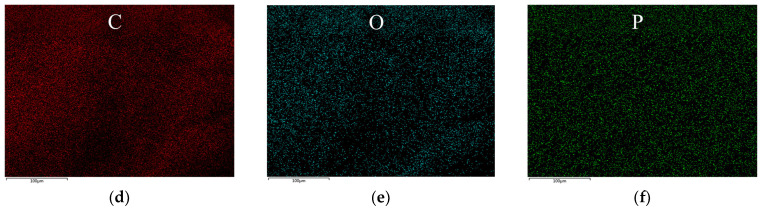
EDS mapping images of (**a**–**c**) EP/DDM and (**d**–**f**) EP/DDM/VDPD-0.6 (elements: C, O, and P).

The SEM observations of the char residues after the combustion of EP/DDM and EP/DDM/VDPD-0.6, shown in Figure 15a,b, demonstrate that the EP char residues were smooth and flat, with only a small number of cracks being generated due to rapid cooling after stopping the combustion, and no outward protruding pores formed due to gas release from the inside to the outside. In contrast, Figure 15c,d show that the char residues of EP/DDM/VDPD-0.6 exhibited pores and honeycomb structures, as well as typical gas-jetting protrusions. The EDS elemental mapping of the char residues obtained from the EP/DDM and EP/DDM/VDPD-0.6 is presented in Figure 16.

**Figure 17:**
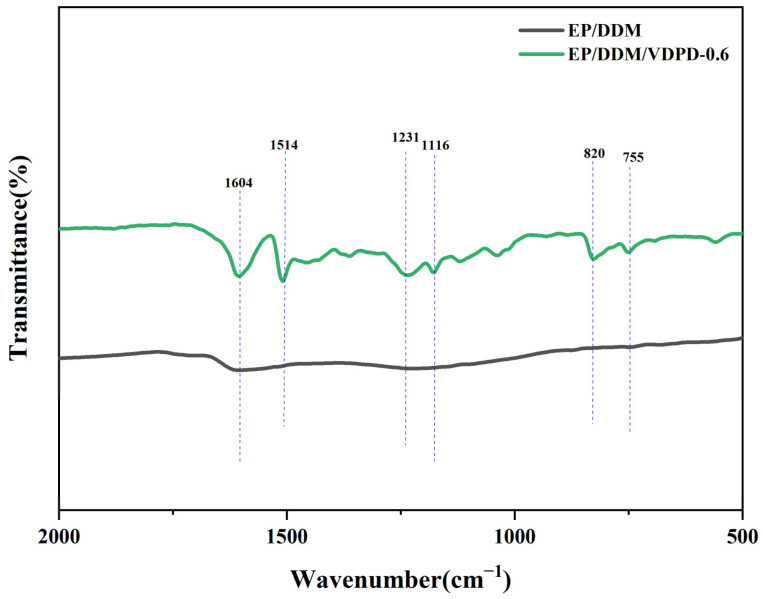
FTIR spectra of char residues of EP/DDM and EP/DDM/VDPD-0.6.

For further confirmation of the composition of the char residues, FTIR spectra were measured, and they are shown in Figure 17. The peaks observed in the spectrum at 1604 cm^−1^ and 1514 cm^−1^ can be assigned to the presence of an aromatic ring. Additionally, the distinct peaks at 1231 cm^−1^ and 1116 cm^−1^ in the spectrum of EP/DDM/VDPD-0.6 correspond to the P=O and P-O-CAr functional groups, respectively. The peaks at 820 cm^−1^ and 755 cm^−1^ are associated with P-O-P and P-O-Ph, respectively. Wang et al. [37] used FTIR to study the thermo-oxidative degradation behaviors of flame retardants and epoxy resins with flame retardants at different temperatures. They detected the formation of P-OH bond stretching vibrations in the FTIR of the flame retardants, so they considered the idea that phosphoric acid derivatives had formed. In addition, when studying the FTIR of the epoxy resins with flame retardants, a new P-O-CAr bond formed, which indicated that DOPO produced phosphoric acid, polyphosphoric acid, and phosphoric acid derivatives during thermal degradation. These phosphoric acids are acidic and react with the decomposed matrix through a dehydration reaction. The presence of P-O-CAr in the char layer of VDPD indicates that a phosphoric acid derivative formed in VDPD and reacted with the decomposed matrix. Moreover, the presence of P-OPh confirmed the existence of phosphorus-containing fragments generated by the decomposition of DOPO groups in the condensed phase during combustion, which indicates that VDPD had a flame-retardant effect in the condensed phase.

**Figure 18:**
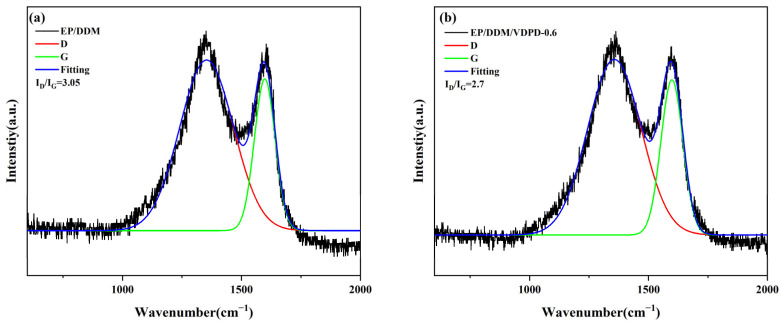
Raman spectra of char residues of EP/DDM (**a**) and EP/DDM/VDPD-0.6 (**b**).

Raman spectroscopy is a common and effective method for characterizing the microstructure of char residues after combustion. The ratio of D and G bands (I_D_/I_G_) in the spectrum can be used to evaluate the graphitization of char residues. Figure 18 presents the spectra of EP/DDM and EP/DDM/VDPD-0.6 after cone calorimeter testing. For EP/DDM, the I_D_/I_G_ ratio was 3.05, but, in the case of EP/DDM/VDPD-0.6, the I_D_/I_G_ ratio dropped to 2.7, decreasing by about 10%. The I_D_/I_G_ ratio is known to be inversely proportional to the in-plane micro-crystallinity of char residual content. Therefore, after combustion, a graphite protective layer was formed by EP/DDM/VDPD-0.6. This protective layer did not produce flames when heated at high temperatures in the air, and it provided better protection during combustion, thereby enhancing the flame-retardant effect. Overall, the introduction of VDPD enhanced the flame-retardant properties in the condensed phase by forming a phosphorus-containing char layer.

### 3.7. Analysis of Pyrolysis Behavior

**Figure 19:**
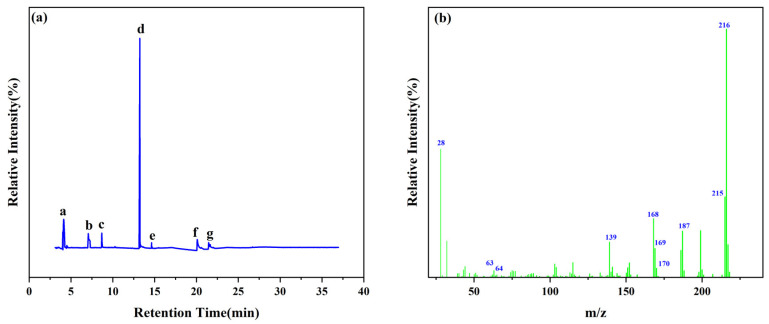
Total ion chromatogram (**a**) and typical MS spectrum of VDPD (**b**).

The thermal decomposition products of VDPD were analyzed using PY/GC-MS to explain the gas-phase combustion mechanism. A total ion chromatogram of VDPD, corresponding to seven decomposition products, namely, toluene (*m*/*z* = 90), benzonitrile (*m*/*z* = 103), guaiacol (*m*/*z* = 124), vanillin (*m*/*z* = 152), 2-phenylphenol (*m*/*z* = 170), benzo guanidine amine (*m*/*z* = 187), and DOPO (*m*/*z* = 216), is depicted in Figure 19a and Table 9. Due to the thermal decomposition of the DOPO group, the presence of diphenyl (*m*/*z* = 154), dibenzofuran (*m*/*z* = 168), and 2-phenylphenol (*m*/*z* = 170) was also observed. Among them, dibenzofuran was produced by the cleavage of the DOPO group, whereas o-phenylphenol was formed by the hydrolysis and cleavage of the P-C bond in the DOPO group.

**Figure 20:**
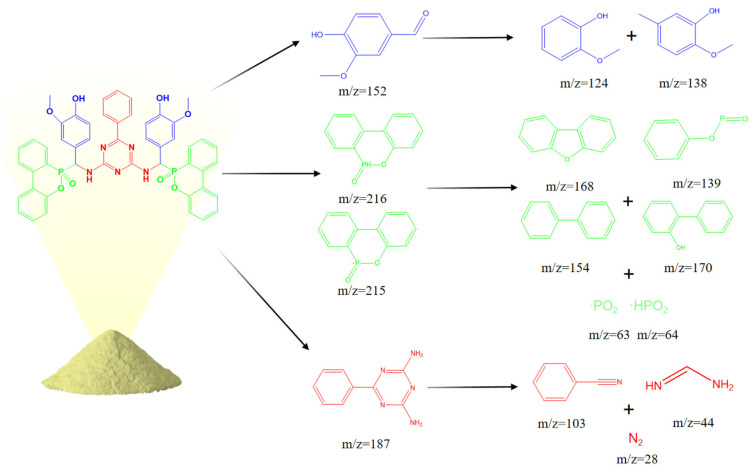
The pyrolysis route of VDPD.

For further analysis of the flame-retardant mechanism of VDPD in the gas phase, the thermal decomposition pathway of VDPD was divided into three parts, as shown in Figure 19b and Figure 20: the first part involves the stepwise degradation of 2-methoxy-5-methylphenol to phenol derivatives, such as 2-methoxyphenol (*m*/*z* = 124) and phenol (*m*/*z* = 93) [13]; the second part consists of the cleavage of benzo guanidine amine (*m*/*z* = 187) into benzonitrile (*m*/*z* = 103) and formamidine (*m*/*z* = 44), which further decompose into non-combustible gases, such as N_2_ (*m*/*z* = 28) [43]; the third part involves the pyrolysis of the phosphaphenanthrene segment into DOPO (*m*/*z* = 216) and DOPO radicals (*m*/*z* = 215), which are then broken down into diphenyl (*m*/*z* = 154), dibenzofuran (*m*/*z* = 168), 2-phenylphenol (*m*/*z* = 170), O=P-O-Ph free radicals (*m*/*z* = 139), •PO_2_ (*m*/*z* = 63), and •HPO_2_ (*m*/*z* = 64) [44]. It could therefore be concluded that, in the gas phase, VDPD acts by producing phosphorus-containing radicals, •PO_2_ and •HPO_2_. These radicals are capable of further reacting with radicals such as •H and •OH. As the triazine group breaks down, nitrogen-containing fragments of inert gas are released, further diluting the combustible gas concentration and expanding the char layer to suppress EP combustion. This further illustrates the flame-retardant effect of the triazine and phosphaphenanthrene groups in EP.

### 3.8. Flame-Retardant Mechanism

**Figure 21:**
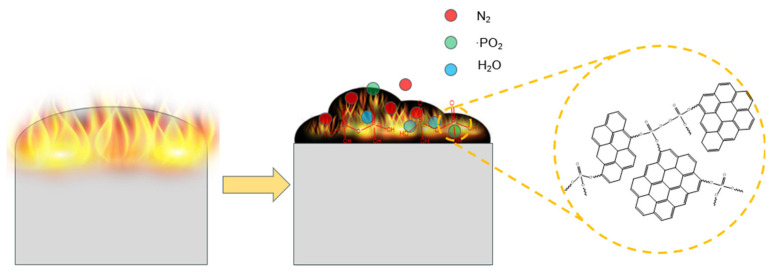
A model of the flame-retardant mechanism of FREP.

By combining the conclusions obtained from the different analysis methods, a flame-retardant mechanism was proposed and is shown in Figure 21. In the gas phase, the triazine group releases nitrogen-containing gas to dilute the degree of combustible gas and form an expansion layer. Phosphorus-containing radicals, such as •PO_2_ and •HPO_2_, are released by the phosphaphenanthrene group. These radicals can react with free radicals, such as •H and •OH, to inhibit EP combustion. In the condensed phase, VDPD decomposes to produce phosphoric acid, polyphosphoric acid, and phosphoric acid derivatives; these phosphoric acids have strong acidity, and they react with the decomposed matrix through a dehydration reaction, thereby promoting the formation of char residues. This physical barrier prevents the further combustion of the EP by effectively preventing heat transfer and the release of combustible gases/smoke [28]. In summary, EP obtains excellent flame-retardant properties with the addition of VDPD, and it benefits from the combined effect of the triazine and phosphaphenanthrene groups in the flame retardant.

## 4. Conclusions

In summary, in this study, a triazine-ring-containing DOPO-derived compound (VDPD) was prepared using VN, DPT, and DOPO and a one-pot method, and it was added to epoxy resin. The curing properties analyzed via DSC demonstrated that VDPD participated in the curing reaction. Although the addition of VDPD resulted in a decrease in T_5%_ and T_max,_ it enhanced the stability of EP at high temperature and promoted char formation. Increasing the VDPD content improved the EP’s flame retardancy. When the P content was increased to 0.4 wt.%, the LOI value of EP/DDM/VDPD-0.4 increased from 25.4% to 33.2%, achieving the V-0 level of UL-94; the PHRR decreased by 32%; and the THR and TSP decreased. PY-GC/MS, LRS, SEM, and FTIR tests revealed the flame-retardant mechanism of VDPD: the triazine group produced a non-combustible gas to dilute the concentration of combustible gas and expand the char layer, and the phosphaphenanthrene group had a quenching effect and promoted the formation of a char layer. Moreover, the addition of VDPD maintained the mechanical properties of the EP. Compared with the epoxy resin without VDPD, the tensile strength of EP/DDM/VDPD-0.4 decreased from 80.4 MPa to 74.3 MPa, only decreasing by 6 MPa, and the tensile modulus increased. Therefore, VDPD is expected to become an epoxy thermosetting material for use in coatings and electronic applications, with broad application prospects.

## Figures and Tables

**Table 1:** Chemical composition of flame—retardant EP.

Sample	E-51 (g)	DDM (g)	VDPD (g)	P (wt.%)
EP	100	25	0	0
EP/DDM/VDPD-0.2	100	25	3.79	0.2
EP/DDM/VDPD-0.4	100	25	7.81	0.4
EP/DDM/VDPD-0.6	100	25	12.13	0.6

**Table 2:** Elemental analysis of VDPD.

Element	C	N	H
Theory (%)	66.29	7.89	4.43
Test (%)	66.26	7.74	4.58

**Table 3:** The E_α_ of EP/DDM and EP/DDM/VDPD-0.6 obtained with the Starink equation.

α	E_α_ of EP/DDM/(kJ/mol)	E_α_ of EP/DDM/VDPD-0.6 (kJ/mol)
0.1	46.58	41.37
0.2	47.86	41.49
0.3	47.65	42.41
0.4	48.76	43.51
0.5	49.46	43.89
0.6	50.19	44.56
0.7	51.29	42.56
0.8	51.57	43.60
0.9	52.25	44.47
Average value	49.51	43.10

**Table 4:** Thermomechanical properties of EP/DDM and EP/DDM/VDPD.

Sample	T_g_ (°C)	E’ at 50 °C (MPa)	E’ at (T_g_ + 40) °C (MPa)	υe (mol/m^3^)
EP/DDM	158	1410	42.3	3600
EP/DDM/VDPD-0.2	155	1460	40.1	3435
EP/DDM/VDPD-0.4	152	1500	37.9	3267
EP/DDM/VDPD-0.6	146	1557	34.3	2996

**Table 5:** Tensile and impact properties of EP and EP/DDM/VDPD.

Samples	Tensile Strength (MPa)	Tensile Modulus (MPa)	Impact Strength (kJ/m^2^)
EP/DDM	80.2 ± 1.4	3077 ± 81	22 ± 0.5
EP/DDM/VDPD-0.2	78 ± 2	3360 ± 65	22 ± 0.4
EP/DDM/VDPD-0.4	74.3 ± 1.6	3446 ± 51	21 ± 0.4
EP/DDM/VDPD-0.6	68.8 ± 1.5	3490 ± 60	21 ± 0.2

**Table 6:** Thermal stability parameters of EP/DDM and EP/DDM/VDPD.

Sample	T_5%_ (°C)	T_max_ (°C)	R_max_ (%/min)	Char Residue at 800 °C (%)
EP/DDM	343	383	16.27	12.22
EP/DDM/VDPD-0.2	328	378	11.17	13.92
EP/DDM/VDPD-0.4	322	375	11.21	15.29
EP/DDM/VDPD-0.6	324	374	9.99	21.20

**Table 7:** LOI and UL-94 test results of five samples.

Sample	LOI (%)	UL-94		Dripping	Level
		t_1_ (S)	t_2_ (S)		
EP/DDM	24.4	--	--	NO	--
EP/DDM/VDPD-0.2	29.5	5.3	6.5	NO	V1
EP/DDM/VDPD-0.4	33.1	4.0	5.2	NO	V0
EP/DDM/VDPD-0.6	34.5	1.2	3.3	NO	V0

**Table 8:** Cone calorimetry test results of four samples.

Sample	PHRR (kW/m^2^)	THR (MJ/m^2^)	SPR (m^2^/s)	av-EHC (MJ/kg)	av-COY (kg/kg)	av-CO_2_ (kg/kg)	Residue (%)
EP/DDM	1679	124.6	0.296	39.70	0.1356	3.42	10.3
EP/DDM/VDPD-0.2	1514	111.4	0.285	36.64	0.1609	3.12	14.5
EP/DDM/VDPD-0.4	1167	103.5	0.261	33.50	0.1887	3.07	15.0
EP/DDM/VDPD-0.6	1265	101.8	0.249	33.29	0.2164	2.84	18.3

**Table 9:** Pyrolysis products identified in the programs of VDPD.

Peak	Retention Time (min)	*m*/*z*	Assigned Structure
a	3.72	91	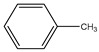
b	7.06	103	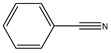
c	8.69	124	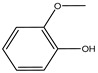
d	13.1	151	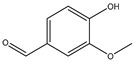
e	14.6	170	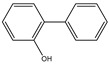
f	20.1	187	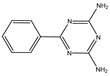
g	21.46	216	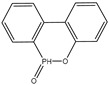

## Data Availability

Data are contained within the article.
